# Supplementary material for: Have Bilateral Free Trade Agreements (BFTAs) been beneficial? Lessons learned from 11 U.S. BFTAs between 1992 and 2017
Source: PLoS One. 2022 Apr 11;17(4):e0264730. doi: 10.1371/journal.pone.0264730 (PMC9000109; doi:10.1371/journal.pone.0264730)
Supplement: Attachment — Submitted filename: Comments_plos one.docx [file pone.0264730.s001.docx]

***Assessment of Bilateral Free Trade Agreements (BFTAs): Lessons from the Existing Twelve U.S. BFTAs between 1992 and 2017***

**Comments**

The manuscript describes the issue of the performance of Bilateral Free Trade Agreements (BFTA)between the United States and twelve nations using a multi-dimensional approach. The issue is important and addresses a research question that matters for the impact on the trading behavior of the United States.

However, it could be improved if the impact of the empirical findings is linked better with the research questions and the extant empirical finding in the literature. This article describes the lack of literature review and opinions, especially in the introduction. It is recommended to restructure and update the latest article or use the literature that matches the data.

Some of the most important specific Comments:

**Abstract**

The author should explain how the role of BFTA in the impact on trade restrictions differs from with existing literature. The abstract should be extended and provide broader insight into the paper.

**Introduction**

In the introduction, there is little mention opinions of the impact of BFTA from the existing literature.

Methodology and results

It is essential to better describe the methodology for readers. Description of results is less connected with the difference from countries, not clearly corresponding with methodology and variables. I strongly recommend authors adjust the presentation of the table. Also, I suggest that authors can do the robustness test, as well as the industry- and country-level tests, before and after the free trade agreements tests, the multilateral trade resistance effect, and crisis, contagion, and BFTA spillover.

Conclusions, Implications and limitations

The implications of the results carried out, the limitations and the future lines of research are less.

Here are my general comments about the paper:

1. Firms were chosen from twelve countries. However, what specific country that these countries were chosen is not clear. Global manufacturing has changed the way U.S. multinational companies make investment decisions, and FTA partners have a large number of production sharing activities in the manufacturing industry. Therefore, the choice of country is important to understand the proportion of BFTA and how these changes throughout the period studied would be an important observation.
2. We suggest authors use the Figure to show the trends of aggregated imports and exports to BFTA countries from the U.S. by trade flow types.
3. Each country in the observation has been affected by the BFTA at different levels. In my opinion, observing the impact of the Log-Real GDP on these countries may lead to biased results in terms of the effect of preferential tariff reductions and tariff margins. I suggest that the effect of agreements will need to adjust for the endogeneity of BFTA.
4. The author's explanation of the possibility of the result is reasonable. However, it is not easy to find from the empirical results that it contains the variables used. For example, the author mentioned that Political influence and intellectual property rights are both factors that lead to the success of BFTA. Therefore, if there are similar findings empirically, it may highlight the contribution.
5. According to Baier & Bergstrand (2007), the presence of the endogeneity may lead to the debate for stable estimates, that is, it may be biased, and the effects of FTAs on trade may be over-or under-estimated. However, it is not a clear discussion or conjecture of explanations in this study.
6. Robustness test applied to OLS for validity and reliability of the study is not clear. The dataset may be divided into before, and after the BFTA, and the period of crisis such as the US-China trade war or the Covid-19 pandemic; that is, for a view of policy perspective for a regional power within the free trade area.

Best of Luck

Reference

Leung, J. Y. (2016). Bilateral vertical specialization between the US and its trade partners—before and after the free trade agreements. *International Review of Economics & Finance*, *45*, 177-196.

Baier, S. L., & Bergstrand, J. H. (2007). Do free trade agreements actually increase members' international trade?. *Journal of international Economics*, *71*(1), 72-95.
